# The ESR Essentials for the medical imaging community

**DOI:** 10.1007/s00330-024-10588-9

**Published:** 2024-02-19

**Authors:** Marc Dewey

**Affiliations:** 1 Department of Radiology, Charité – Universitätsmedizin Berlin, corporate member of Freie Universität Berlin and Humboldt-Universität zu Berlin, Berlin, Germany; 2 Berlin Institute of Health at Charité – Universitätsmedizin Berlin, Berlin, Germany; 3 Berlin University Alliance, Berlin, Germany; 4 DZHK (German Centre for Cardiovascular Research), Partner Site Berlin, Deutsches Herzzentrum der Charité (DHZC), and DKTK (German Cancer Consortium), partner site Berlin, Berlin, Germany

Clinical research in imaging is at an inflection point. We need to move beyond small single-center observational studies to multi-center medical imaging trials [1, 2]. This is illustrated by the upcoming 10th anniversary of the Clinical Trials in Radiology (CTiR) sessions at the European Congress of Radiology (ECR) in 2024 (https://www.myesr.org/congress/). Randomized medical imaging trials have a substantial impact on clinical practice by prompting changes in the use of medical imaging technologies for specific clinical scenarios.

Changing clinical medicine depends on three crucial factors: first, new evidence primarily derived from robust data generated from randomized trials or large registries; second, reimbursement decisions by health technology assessment agencies based on clinical evidence and health economics studies; and third, a general consensus on the application of diagnostic tests and therapy within medical communities. Achieving consensus in the community often relies on acceptance of updated or revised clinical practice guidelines.

Clinical guidelines often tend to be long and cater to the needs of medical subspecialties. To drive change in medical imaging, it is crucial to provide the general medical imaging community with straightforward, evidence-based recommendations for clinical practice.

We are therefore pleased to introduce a new series, the ESR Essentials, that has been developed in close collaboration with the European medical imaging subspecialty societies, which have selected the authors of the respective writing groups and decided on the topics of the articles in agreement with the guest editor of the Essentials. The Essentials is a series of both concise and evidence-based practice recommendations that summarize the core knowledge for pivotal questions in clinical care and are aimed at a general radiologist audience. The vision is to inform the community of best-practice imaging supported by the highest evidence in relevant topics by each of the subspecialty societies.

We hope that the Essentials will provide a more accessible knowledge base and more commonly used guide to clinical recommendations owing to their concise but evidence-based nature. Importantly, all 14 European subspecialty medical imaging societies have agreed to join forces with the ESR, the *European Radiology* Editor and Editorial Board, the Editorial Office, the Guest Editor, and the ESR Publications Committee in bringing to our readers this new format.

Almost all Essentials articles feature several key aspects that aim to enhance comprehensibility and direct usability within clinical practice. First, the articles will be succinct, and second, they will be composed by the foremost experts in their field. Third, the Essentials will specify the current levels of evidence [3] and whether the underlying studies were conducted at a single or multiple centers (presented in tables) for every significant recommendation made. Fourth, they will make use of flowcharts of the clinical pathways to summarize recommendations and provide specific guidance for clinical situations. Fifth, the Essentials will provide exquisite illustrations of the typical imaging results that can be expected along common clinical pathways.

I am confident that the Essentials will aid readers in utilizing medical imaging technologies effectively in specific clinical scenarios. The articles will also account for variations in available equipment and expertise among readers, enabling them to apply the Essentials to their own settings. Subspecialty writing groups have the freedom to obtain input from additional non-imaging specialty societies, including surgical and medical subspecialties, to generate genuine multidisciplinary recommendations. If necessary, specific protocols outlining image acquisition, reconstruction, and analysis can be appended as electronic supplementary material to the articles.

I strongly believe that this initiative will be pivotal in the future of medical imaging. This is because we need to gather more data on important questions that are verifiable and broadly generalizable using global collaboration and data sharing [4]. This may lead to better ways of generating the essentials of radiology for our community and lead the way forward in medical imaging.

In order to actually start to implement this vision, it is crucial that the core clinical information about the use of medical imaging is made available in both a digestible and evidence-based fashion for the entire radiological community to increase the uptake of trial results in clinical practice. The final aim of the ESR Essentials initiative is to enable each subspecialty medical imaging society to publish ten articles, each updated every four to five years, resulting in an overall number of 120–130 articles in this series being available in 2028.

The first two Essentials we are publishing in this issue of *European Radiology* are from the European Society of Gastrointestinal and Abdominal Radiology (ESGAR) and the European Society of Cardiovascular Radiology (ESCR), and are dedicated to diagnosing hepatocellular carcinoma [5] and cardiac MRI [6].

I am immensely thankful for the support and input from the ESR Board of Directors and the ESR Executive Council, the editor of the ESR Journal Family, the editors and editorial board members of *European Radiology*, *Insights into Imaging*, *European Radiology Experimental*, *Eurorad*, and, last but not least, the ESR and editorial office. I wish the writing groups that their Essentials are met with appreciation by the medical imaging community and will help change clinical medicine for the better.

## Declarations

### Guarantor

The scientific guarantor of this publication is Marc Dewey.

### Statistics and biometry

No complex statistical methods were necessary for this paper.

### Methodology

- • Not applicable.
